# Reliability and criterion-related validity with a smartphone used in timed-up-and-go test

**DOI:** 10.1186/1475-925X-13-156

**Published:** 2014-12-02

**Authors:** Alejandro Galán-Mercant, Francisco Javier Barón-López, María T Labajos-Manzanares, Antonio I Cuesta-Vargas

**Affiliations:** Departamento de Psiquiatría y Fisioterapia, Facultad de Ciencias de la Salud, Universidad de Málaga, Andalucia Tech, Cátedra de Fisioterapia y Discapacidad, Instituto de Biomedicina de Málaga (IBIMA), Grupo de Clinimetria (AE-14), Av/ Arquitecto Peñalosa s/n (Teatinos Campus Expansion), 29009 Málaga, Spain; Departamento de Medicina Preventiva, Facultad de Medicina, Universidad de Málaga, Andalucia Tech, Malaga, Malaga, Spain; School of Clinical Sciences of the Faculty of Health at the Queensland, University of Technology, Brisbane, Australia

**Keywords:** Inertial sensor, Smartphone, iPhone, Older adult, Validation

## Abstract

**Background:**

The capacity to diagnosys, quantify and evaluate movement beyond the general confines of a clinical environment under effectiveness conditions may alleviate rampant strain on limited, expensive and highly specialized medical resources. An iPhone 4® mounted a three dimensional accelerometer subsystem with highly robust software applications. The present study aimed to evaluate the reliability and concurrent criterion-related validity of the accelerations with an iPhone 4® in an Extended Timed Get Up and Go test. Extended Timed Get Up and Go is a clinical test with that the patient get up from the chair and walking ten meters, turn and coming back to the chair.

**Methods:**

A repeated measure, cross-sectional, analytical study. Test-retest reliability of the kinematic measurements of the iPhone 4® compared with a standard validated laboratory device. We calculated the Coefficient of Multiple Correlation between the two sensors acceleration signal of each subject, in each sub-stage, in each of the three Extended Timed Get Up and Go test trials. To investigate statistical agreement between the two sensors we used the Bland-Altman method.

**Results:**

With respect to the analysis of the correlation data in the present work, the Coefficient of Multiple Correlation of the five subjects in their triplicated trials were as follows: in sub-phase Sit to Stand the ranged between r = 0.991 to 0.842; in Gait Go, r = 0.967 to 0.852; in Turn, 0.979 to 0.798; in Gait Come, 0.964 to 0.887; and in Turn to Stand to Sit, 0.992 to 0.877. All the correlations between the sensors were significant (p < 0.001). The Bland-Altman plots obtained showed a solid tendency to stay at close to zero, especially on the *y* and *x*-axes, during the five phases of the Extended Timed Get Up and Go test.

**Conclusions:**

The inertial sensor mounted in the iPhone 4® is sufficiently reliable and accurate to evaluate and identify the kinematic patterns in an Extended Timed Get and Go test. While analysis and interpretation of 3D kinematics data continue to be dauntingly complex, the iPhone 4® makes the task of acquiring the data relatively inexpensive and easy to use.

## Background

The human movement evaluation is considered to be a cornerstone of both the generation of knowledge and the assessment of the effect of clinical treatments [1]. The most commonly technique of human movement evaluation used in physical therapy clinical practice has been the one-dimensional movable-arm goniometry [1–3]. The devices used in this clinical practice, however, provide information on motion in just a single plane [3]. Laboratory systems designed to measure more dimensions and with greater precision are complex and expensive [1]. The commonest are electromagnetic and optoelectronic video systems [4]. A major drawback of the electromagnetic devices is that they may be adversely affected by the presence of metals [5], and they are limited to the area covered by the source of the magnetic field and by the wiring connecting the sensors to the source. Optoelectronic systems, too, present serious limitations due to their complexity and the time needed to perform the entire process [6]. Currently, new motion analysis devices are being developed that are smaller, lighter, portable, and precise, so as to offer a better alternative for the measurement of patterns of movement in a practical clinical setting [4].

The latest generation of smartphones often incorporate micro-electromechanical inertial systems with accelerometers and gyroscopes that can detect acceleration and measure for rate Cobb Angles [7], functional balance tests or tasks such as Timed Get Up and Go Extended [8], Sit-to-Stand and Stand-to-Sit transitions [9] or analysing frail older adults during a turn transition [10]. There are applications available which can read, store, transfer, and display data from the accelerometer and gyroscope, endowing these smartphones with an enormous potential for monitoring the parameters of human movement in general, and for clinical use in particular.

The objective of the present study was to evaluate the reliability and concurrent criterion-related validity of the measurements of acceleration in each of the three axes of motion as obtained from an iPhone 4® during the performance of an *Expanded Timed-Get-Up-and-Go* (ETGUG) test.

## Methods

### Design and participants

A cross-sectional, repeated measure, analytical study was designed to examine the intra-individual reliability and concurrent criterion-related validity of the smartphone iPhone 4® in the ETGUG test. Five participants were recruited from a healthy over-65 population. The exclusion criteria were a history of pain in the last twelve months, or a history of surgery, malignancy, or musculoskeletal disorders of any limb that might be aggravated by the procedures involved in the test. Informed consent was obtained from all subjects, and study procedures were consistent with the Helsinki declaration. The study was approved by the local University Committee.

### Inertial sensors

The participants wore the two sensors overlapping in a small neoprene sleeve, placed at the level of the middle third of the sternum. Previous studies on the variability of inertial sensor measurements placed at the levels of different body segments [11, 12] show that sensors located at the level of the sternum provide reliable data. The first of the sensors used was an Inertiacube3® (IC3; Intersense, Bedford, MA, USA). This module integrates two two-axes accelerometer, three singles-axis gyroscopes and a three-axis magnetometer compass within low volume (26.2 × 39.2 × 14.8 mm^3^). InertiaCube3 combines the aforementioned sensing elements with an integrated Kalman filtering algorithm. The unit can provide orientation and gravity compensated acceleration information aligned with the Earth’s magnetic north. InertiaCube3 can measured accelerations up to ±6 g [13], in this study were studied only the values obtained from the accelerometer subunit.

The second sensor (the one to be validated in the present study) was that incorporated in the iPhone 4® of Apple®. This smartphone is equipped, as is the IC3, with three triaxial elements for the detection of kinematic variables: a gyroscope, a magnetometer, and an accelerometer. Apple uses an LIS302DL accelerometer in the iPhone 4® [14]. Kinematic data were acquired using the xSensor® Pro software of Crossbow Technology, Inc. This app couldn’t record at higher sampling rates than 32 Hz. A previous study [7] had validated the iPhone 4®’s gyroscope, showing the inter-observer error to be 4.0° (standard deviation of the difference between measurements).

The orientation and movement of these two sensors are presented as Euler angles RPY (roll, pitch, and yaw). If the sensor’s RPY axes are aligned with the anatomical axes of the trunk, the roll angle of a movement is around the anteroposterior (AP) axis [15]. Movements in this plane are less frequent than those in other planes [16]. The pitch angle is around the left-right (or mediolateral, ML) axis [15], and the yaw angle is around the vertical (V) axis [15].

### Test protocol

The subjects performed the ETGUG test three times. They used a chair without armrests, and were instructed orally not to use their arms to stand up or sit down. Various studies have explored this test using chairs without armrests [17–19]. This choice could reduce inter-subject variability by eliminating the option of whether or not to use the arms in the standing up and sitting down phases. The ETGUG test selected was that using a 10 metres long corridor, the aim being to include as many gait cycles during the test as possible [19]. The beginning and end of the ten metres were marked on the floor with 2½-cm wide tape. The protocol was as follows:

1. The subject sat with his or her back in contact with the back of the chair (the seat was 460 mm high and lacks armrests).
2. The ETGUG begins with the therapist’s go sign and the subject stands up (Sit-to-Stand).
3. The subject begins walking ten meters (Gait Go).
4. The subject turns around a wide tape mark placed 10 m away from the chair (Turn).
5. The subject walks back toward the chair (Gait Come).
6. The subject turns away from the chair to sit down (Turn-to-Stand-to-Sit).

To evaluate the test-retest reliability of the iPhone 4®’s kinematic measurements, the participants repeated the protocol three times. The first and second trials were completed consecutively for reliability and after the two sensors were removed from the participant’s body following an hour of rest, the sensors were put back in the same position, and the ETGUG test protocol was repeated to evaluate the stability of the measurement after removing and then putting back the two sensors. It was assumed that the participant’s performance remained unchanged within the time of this resting period. The same examiner used the same device and the same protocol to place the devices, and to conduct the ETGUG test.

### Sub-phases of the expanded timed-get-up-and-go test

**Figure 1:**
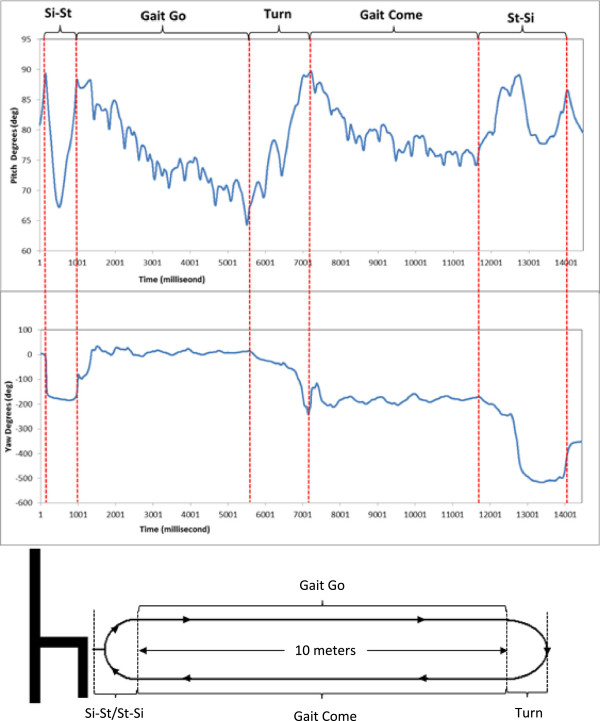
**Accelerometry identification of the ETGUG components – representation of the pitch and yaw signals (in degrees).**

After the test, and based on the analysis of the accelerometry data of all the participants, the ETGUG test was divided into 5 main sub-stages: from sitting to standing (Sit-to-Stand, Si-St), gait going away (Gait Go, GG), turn (T), gait coming back (Gait Come, GC), and turning to sit down (Turn-to-Stand-to-Sit, T-St-Si) (Figure 1). The different sub-phases were detected with the sensor parameters as follows:

- For the identification and analysis of the sitting-standing transitions (Si-St and T-St-Si), we followed a previously published protocol [20] (Figure 1).
- For the identification and analysis of the turn transition (T), we also followed a previously published method [21] in which 180° rotations are detected by analysing the yaw rotation signal, which should identify the first turn at ten metres and the second turn which is made in order to sit down and thus terminate the test (Figure 1).
- The identification and analysis of the GG and GC sub-phases was performed by analysing the data remaining once the Si-St and T-St-Si [20] and T [21] sub-phases had been detected and delimited.

### Signal processing

The IC3 data were acquired at a 100 Hz sampling frequency, and those of the iPhone 4® at 32 Hz. Data processing was performed off-line, expanding and synchronizing the two sets of time series using the basic package of the R® software environment.

### Statistical analysis

We performed descriptive statistics with measures of central tendency and dispersion of the maximum and minimum peak acceleration in AP, ML, V and the acceleration magnitude (AM). The AM is a vector that has the same effect on the system as the component vectors. AM is calculated from the three acceleration vectors of the axes of motion (x, y, z) as the square root of the sum of the values of the three axes (AM = √*x*^2^ + *y*^2^ + *z*^2^). Analyses of the relations between the scores obtained by simultaneously from IC3 and iPhone 4® were performed. The relations were studied by using the Coefficient of Multiple Correlation (CMC) [22]. Cohen and Holiday criteria [23] were applied to interpret these correlation coefficients, suggesting the following categorization: very low correlation for values below 0.20; low correlation for values between 0.20 and 0.39; moderate correlation for values between 0.40 and 0.69; high correlation for values between 0.70 and 0.89 and very high correlation for values above 0.89. To evaluate the reliability of the iPhone 4® measurements, we calculated the intraclass correlation coefficient (ICC) between the two sensors acceleration signal. Values between 0.70 and 0.95 were considered acceptable reliability indicators [23]. To investigate statistical agreement between the two sensors we used the Bland-Altman method [24].

## Results

**Figure 2:**
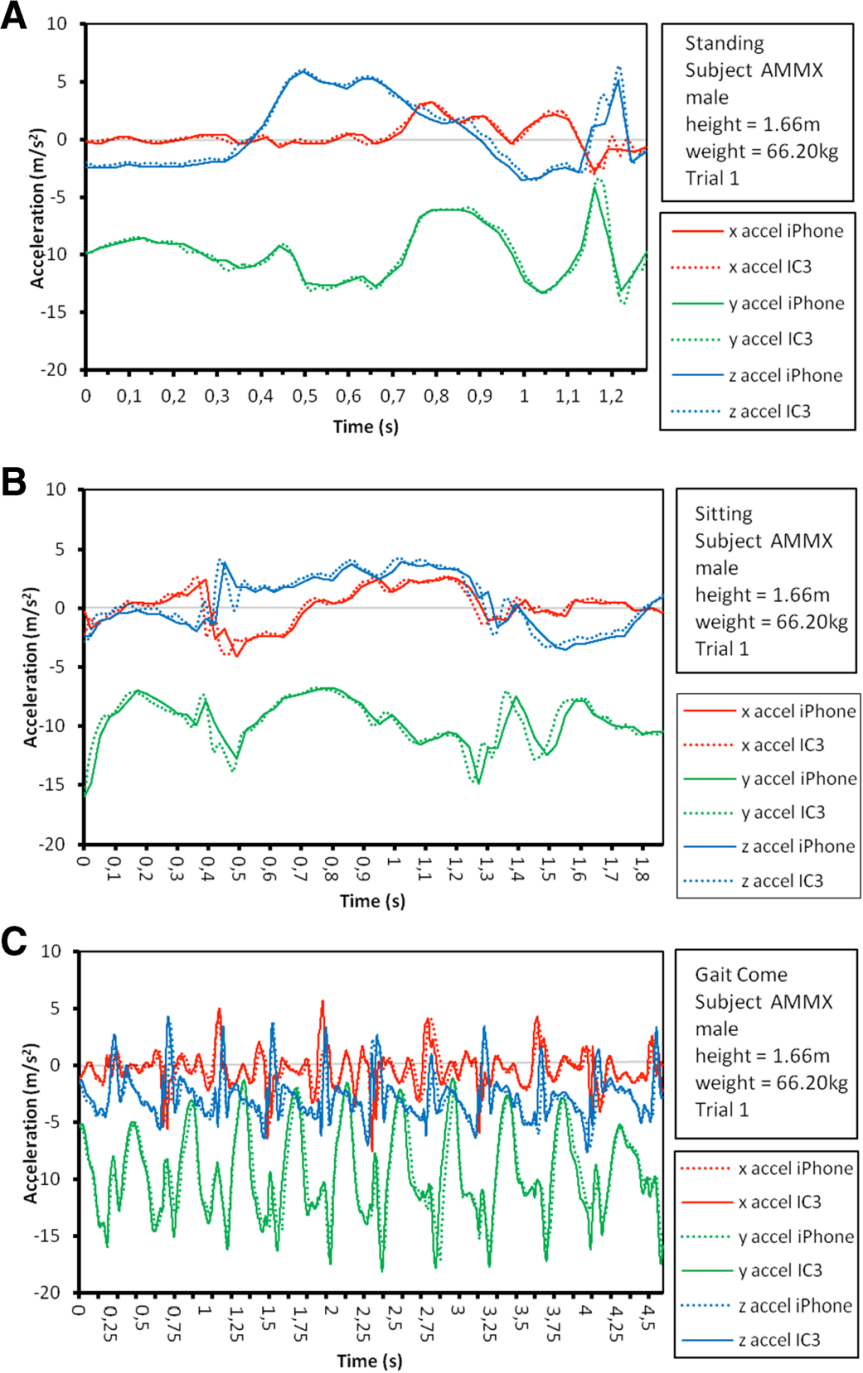
**Graphical illustration of the acceleration values in the different sub-phases of the ETGUG test. A**. Accelerations in the standing phase. **B**. Accelerations in the sitting phase. **C**. Accelerations in the gait come phase.

**Figure 3:**
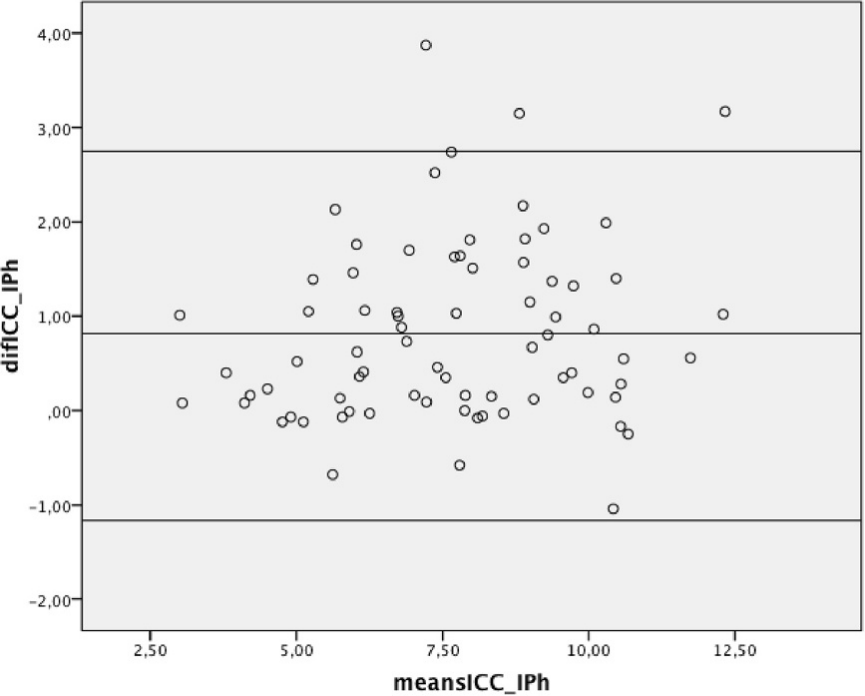
**Bland-Altman plot for acceleration magnitude of motion, for the GG sub-phase with two devices.**

Table 1 presents the descriptive statistics of the anthropometric information of the five participants in the study, and their scores in times for each of the sets of three ETGUG trials. The two sets of ETGUG test kinematic data for the iPhone 4® and IC3, maximum and minimum peaks, for the AP, ML, V and the AM are summarized in Table 2. The results of the CMC analysis between the resultant vectors of the two time series in the different sub-phases of the ETGUG test are presented in Table 3. All the correlations between the sensors were significant (p < 0.001). The CMC of the five subjects in their triplicated trials were as follows: in sub-phase Si-St the ranged between r = 0.991 to 0.842; in GG, r = 0.967 to 0.852; in T, 0.979 to 0.798; in GC, 0.964 to 0.887; and in T-St-Si, 0.992 to 0.877 (Table 3). Table 4 presents the Intra-class correlations (ICC) for IC3 peak acceleration and iPhone 4® peak acceleration, the lower value ICC was 0.819 and the higher value was 0.987. Figure 2 shows a graphical example of a subject in which one observes the strong correlation between the sensors, and the high level of agreement between the signals of the two motion sensors in the different sub-phases of the test. The whole 225 worked out Bland-Altman plots obtained showed a solid tendency to stay agreement, Figure 3 shows the Bland-Altman plot of GG sub-phase of the ETGUG test, in which one can appreciate the degree of agreement between the two sets of data (acquired from the iPhone 4® and the IC3) for AM of motion.

**Table 1:** **Anthropometric data and ETGUG total time score (** ***N*** **= 5)**

Subject	Age (years)	Weight (kg)	Height (cm)	BMI (kg/m ^2^)	ETGUG Trial 1(s)	ETGUG Trial 2(s)	ETGUG Trial 3(s)
**Subject 1**	67	66.20	166	24.02	12.96	13.60	13.33
**Subject 2**	72	74.50	172	25.18	12.02	11.65	11.34
**Subject 3**	67	102.30	175	33.40	12.04	12.22	11.19
**Subject 4**	69	86.00	176	27.76	11.76	11.78	12.16
**Subject 5**	65	79.00	166	28.67	11.71	11.13	11.45
**Mean ± SD**	**68 ± 2.65**	**81.60 ± 13.62**	**171 ± 4.70**	**27.81 ± 3.65**	**12.09 ± 0.51**	**12.08 ± 0.94**	**11.89 ± 0.88**

*Kg*. Kilograms; *cm*. Centimetres; *m*. Metres; *s*. Seconds.

**Table 2:** **Kinematic data of sample (** ***N*** **= 5)**

PA (m/s ^2^) ± SD	Standing	Gait Go	Turn	Gait Come	Siting
**AP max IC3 (m/s** ^**2**^ **± SD)**	7.54 ± 2.41	4.45 ± 2.16	3.97 ± 2.68	3.66 ± 1.42	5.64 ± 1.79
**AP max iPh (m/s** ^**2**^ **± SD)**	7.14 ± 2.39	3.51 ± 1.93	2.60 ± 1.62	2.27 ± 1.12	5.16 ± 1.757
**AP min IC3 (m/s** ^**2**^ **± SD)**	−5.17 ± 1.89	−7.23 ± 1.54	−6.52 ± 2.61	−7.56 ± 1.62	−5.47 ± 2.68
**AP min iPh (m/s** ^**2**^ **± SD)**	−5.12 ± 1.82	−7.01 ± 1.47	5.91 ± 2.11	−7.22 ± 1.16	−5.62 ± 2.62
**ML max IC3 (m/s** ^**2**^ **± SD)**	3.94 ± 1.33	6.54 ± 6.55	3.91 ± 2.07	6.18 ± 2.38	3.59 ± 1.43
**ML max iPh (m/s** ^**2**^ **± SD)**	3.87 ± 1.23	5.94 ± 2.27	3.69 ± 1.81	5.36 ± 1.96	3.53 ± 1.16
**ML min IC3 (m/s** ^**2**^ **± SD)**	−4.81 ± 1.68	−8.33 ± 2.36	−7.64 ± −7.65	−7.73 ± 2.62	−4.61 ± 1.92
**ML min iPh (m/s** ^**2**^ **± SD)**	−4.07 ± 1.23	−7.06 ± 2.17	−7.03 ± 3.27	−6.42 ± 2.93	−4.02 ± 1.81
**V max IC3 (m/s** ^**2**^ **± SD)**	−2.55 ± 1.94	0.60 ± 1.79	−2.23 ± 2.68	0.82 ± 1.96	−3.77 ± 2.37
**V max iPh (m/s** ^**2**^ **± SD)**	−2.93 ± 2.06	−0.005 ± 1.57	2.48 ± 2.51	0.17 ± 1.39	−4.06 ± 2.12
**V min IC3 (m/s** ^**2**^ **± SD)**	−16.43 ± 2.39	−21.62 ± 2.45	−22.79 ± 7.94	−21.88 ± 4.23	−21.07 ± 5.21
**V min iPh (m/s** ^**2**^ **± SD)**	−15.51 ± 1.61	−20.13 ± 1.86	−19.19 ± 2.69	−19.51 ± 2.38	−19.21 ± 3.24
**AM max IC3 (m/s** ^**2**^ **± SD)**	9.24 ± 2.10	8.41 ± 2.52	6.96 ± 2.29	7.55 ± 2.54	8.21 ± 1.41
**AM max iPh (m/s** ^**2**^ **± SD)**	8.99 ± 2.15	7.33 ± 2.22	5.99 ± 1.47	6.07 ± 1.98	7.91 ± 1.23
**AM min IC3 (m/s** ^**2**^ **± SD)**	18.02 ± 2.71	24.37 ± 2.98	25.01 ± 8.59	24.48 ± 4.84	22.37 ± 5.70
**AM min iPh (m/s** ^**2**^ **± SD)**	16.93 ± 1.93	22.56 ± 2.28	21.44 ± 3.83	21.91 ± 3.04	20.56 ± 3.82
**Time (s ± SD)**	1.39 ± 0.26	3.92 ± 0.45	1.99 ± 0.48	4.14 ± 0.41	1.96 ± 0.31

*PA*. Peak acceleration; *AP*. Anteroposterior; *ML*. Mediolateral; *V*. Vertical; *AM*. Acceleration magnitude; *m*. Metres; *s*. Seconds; *SD*. Standard Desviation; *IC3*. Inertial Cube 3; *iPh*. iPhone.

**Table 3:** **Coefficient of Multiple Correlation (CMC) in the different phases of the ETGUG (** ***N*** **= 5)**

Subject (Trial)	Standing	Gait Go	Turn	Gait Come	Siting	Mean ± SD
**Subject 1 (T1)**	0.985	0.951	0.967	0.931	0.877	**0.942 ± 0.04**
**Subject 1 (T2)**	0.951	0.945	0.979	0.962	0.939	**0.955 ± 0.02**
**Subject 1 (T3)**	0.963	0.953	0.968	0.953	0.992	**0.967 ± 0.02**
**Subject 2 (T1)**	0.981	0.958	0.979	0.956	0.974	**0.970 ± 0.01**
**Subject 2 (T2)**	0.954	0.956	0.971	0.956	0.951	**0.954 ± 0.01**
**Subject 2 (T3)**	0.991	0.952	0.974	0.950	0.945	**0.962 ± 0.02**
**Subject 3 (T1)**	0.946	0.872	0.798	0.887	0.961	**0.893 ± 0.06**
**Subject 3 (T2)**	0.906	0.852	0.891	0.874	0.956	**0.896 ± 0.04**
**Subject 3 (T3)**	0.955	0.909	0.957	0.913	0.923	**0.931 ± 0.02**
**Subject 4 (T1)**	0.983	0.931	0.957	0.949	0.975	**0.959 ± 0.02**
**Subject 4 (T2)**	0.986	0.967	0.952	0.924	0.938	**0.953 ± 0.02**
**Subject 4 (T3)**	0.965	0.956	0.976	0.964	0.911	**0.954 ± 0.03**
**Subject 5 (T1)**	0.842	0.952	0.935	0.960	0.978	**0.933 ± 0.05**
**Subject 5 (T2)**	0.969	0.963	0.938	0.956	0.955	**0.956 ± 0.01**
**Subject 5 (T3)**	0.972	0.949	0.864	0.964	0.963	**0.942 ± 0.05**
**Mean ± SD**	**0.967 ± 0.04**	**0.938 ± 0.03**	**0.941 ± 0.05**	**0.939 ± 0.03**	**0.949 ± 0.03**	

*T*. Trial; *SD*. Standard Desviation.

**Table 4:** **Intra-class correlations for IC3 peak acceleration and iPhone 4® peak acceleration**

Peak acceleration	ICC (95%)
**AM maximum**	0.952 (0.925–0.970)
**AM minimum**	0.877 (0.805–0.922)
**ML maximum**	0.967 (0.947–0.979)
**ML minimum**	0.966 (0.946–0.978)
**V maximum**	0.987 (0.980–0.992)
**V minimum**	0.819 (0.714–0.886)
**AP maximum**	0.970 (0.952–0.981)
**AP minimum**	0.968 (0.950–0.980)

*ICC*. Intraclass correlation coefficient (lower bound-upper bound); *AM*. Acceleration magnitude; *AP*. Anteroposterior; *ML*. Mediolateral; *V*. Vertical.

## Discussion

We have investigated the reliability and concurrent criterion-related validity of the acceleration values along each of the three axes of motion acquired from an iPhone 4® during the performance of the ETGUG test by healthy elderly subjects, comparing them with the values acquired simultaneously with a standard validated laboratory device (IC3). There was a strong correlation between the two data sets. ICC is the most appropriate and widely used indicator to test the reproducibility of continuous measures. The results of our study, always above or equal to 0.80, prove that the iPhone 4 accelerations showed good levels of reproducibility. The Bland-Altman plots also showed high levels of agreement. The iPhone 4® thus proved to be reliable and valid for the identification of kinematic patterns in the performance of the ETGUG test by healthy elderly subjects.

To the best of our knowledge, the present study is the first designed to seek evidence for or against reliability and validity of the iPhone 4® as a tool for the analysis and identification of kinematic patterns in the accelerometry data acquired during the performance of the ETGUG test by healthy elderly subjects. A previous study [25] evaluated the implementation of the iPhone 3® as an accelerometer to quantify kinematic variables related to gait. It concluded that the device demonstrated sufficient validity and consistency for the different measurements considered.

However, unlike the present case, that study reports no design information and/or analyses to evaluate the device’s reliability and/or validity. Another study [26] evaluated the implementation of the iPhone 3® as an accelerometer to identify tremors in Parkinson’s sufferers. As in the previous case [25], again the document includes no design information and/or analyses to evaluate the reliability and/or validity prior to the possible characterization and identification of the Parkinsonian tremors reported in the study. Two recent studies [27, 28], evaluated the reliability and validity of a smartphone accelerometer. In both studies, the Smartphone were an Android phone, against the used in the present study (iPhone 4®).

As we presented in the results section, the worked out Bland-Altman plots obtained showed a solid tendency to stay at close to zero, especially on the *y* and *x*-axes, during the five phases of the ETGUG test (Figure 3). Nonetheless, there was a slight tendency to underestimate the data on the *z*-axis during the five phases of the test. The accumulated *z*-axis error never exceeded −0.5 m/s^2^, however (Figure 3). Using the method proposed by Bland & Altman [24], we would conclude that the two sensors are comparable in the measurement of accelerometry variables, since the differences found between the values recorded by the iPhone 4® and IC3 were minimal (Figure 3). Indeed, these differences were so small that they could well have been due to other methodological factors that may have biased the clinical result, such as the movement of the skin or clothing, or misalignment of the sensor.

The main contribution of the present study has been that the demonstration of the high levels of reliability and validity of the iPhone 4® in measuring the kinematic variables of human motion make this device potentially very interesting for eventual use in a clinical setting. To this end, it will be necessary to develop the appropriate software for its use. In the specific case of the analysis of the kinematics of the trunk in the ETGUG test with an iPhone 4®, it would be possible to measure absolute and averaged values of the speed of gait, the angular velocity during turns, acceleration along different axes of motion, acceleration in the different sub-phases of the test, and gait-related variables (cadence, stride length, *etc.*). The identification of the different sub-phases of the test could be performed using algorithms for segmenting the test applied to both devices. Further studies could be analyzed biggest samples and the sensitive of changes after an intervention.

## Conclusions

We conclude that the inertial sensor incorporated in the iPhone 4® has the sufficient reliability and validity for the evaluation and identification of kinematic patterns in the ETGUG test. While analysis and interpretation of 3D kinematics data continue to be dauntingly complex, the iPhone 4® makes the task of acquiring the data relatively inexpensive and easy to use. In particular, it could be used in clinical practice or other environments of interest (such as patients’ homes, specialized centres, *etc.*), unlike other types of inertial sensors (e.g., the IC3) with their high costs and complex signal processing. The evidence clearly points to the suitability of the iPhone for the recognition of patterns of kinematics of the trunk during ETGUG testing, although this application could be extended to other functional activities.
